# Surface Modification, Toxicity, and Applications of Carbon Dots to Cancer Theranosis: A Review

**DOI:** 10.3390/nano15110781

**Published:** 2025-05-22

**Authors:** Tirusew Tegafaw, Endale Mulugeta, Dejun Zhao, Ying Liu, Xiaoran Chen, Ahrum Baek, Jihyun Kim, Yongmin Chang, Gang Ho Lee

**Affiliations:** 1 Department of Chemistry, College of Natural Sciences, Kyungpook National University, Taegu 41566, Republic of Korea; tirukorea@gmail.com (T.T.); endexindex05@gmail.com (E.M.); djzhao.chem@gmail.com (D.Z.); ly1124161@gmail.com (Y.L.); tsukiyovo@gmail.com (X.C.); 2 Institute of Biomedical Engineering Research, Kyungpook National University, Taegu 41944, Republic of Korea; baxun@naver.com; 3 Department of Chemistry Education, Teachers’ College, Kyungpook National University, Taegu 41566, Republic of Korea; jkim23@knu.ac.kr; 4 Department of Molecular Medicine, School of Medicine, Kyungpook National University, Taegu 41944, Republic of Korea

**Keywords:** carbon dot, surface modification, toxicity, cancer, theranosis

## Abstract

Cancer remains one of the leading causes of death worldwide, prompting extensive research into novel theranostic (combined word of diagnostic and therapeutic) strategies. Nanomedicine has emerged as a potential breakthrough in cancer theranosis, overcoming limitations of conventional approaches. Among such approaches, carbon dots (CDs) with a size smaller than 10 nm have garnered significant attention for their potential use in cancer theranosis, owing to their low toxicity, good water solubility, easy synthesis, facile surface modification, and unique optical and photothermal and photodynamic properties. Researchers have demonstrated that surface functionalization of CDs with diverse hydrophilic groups can be easily achieved by choosing proper carbon precursors in synthesis, and further surface modification of CDs with cancer-targeting ligands, photosensitizers, anticancer drugs, and genes can also be easily achieved using various methods, thereby establishing a versatile approach for cancer theranosis. This review described the various surface modification methods of CDs, in vitro and in vivo toxicity of CDs, and various cancer theranostic methods such as drug delivery, photodynamic therapy, photothermal therapy, gene therapy, sonodynamic therapy, and gas therapy. Therefore, CDs can serve as various mono and combined theranostic modalities, offering us new methods for cancer theranosis.

## 1. Introduction

**Figure 1:**
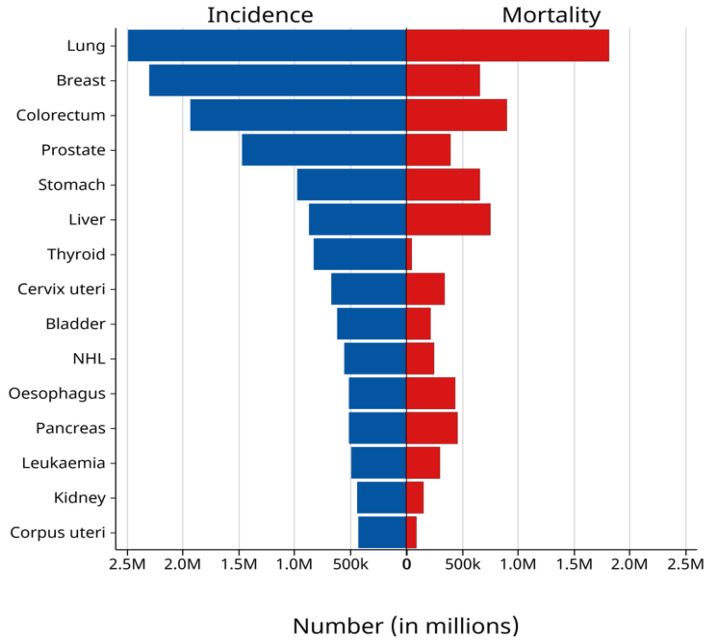
Cancer incidence and mortality worldwide in both sexes and all ages for the year 2022. Graph from Global Cancer Observatory (GLOBOCAN) 2022, International Agency for Research on Cancer (IARC), World Health Organization [2].

Cancer is a major threat influencing the global population and posing a major challenge to human life. According to the most recent report from the International Agency for Research on Cancer (IARC), 20 million new cancer cases were diagnosed globally in 2022, and among them, 9.7 million people died from various forms of cancer worldwide (Figure 1) [1,2]. Considering the high risk and mortality of cancer, early diagnosis is most effective, and the development of innovative treatment strategies is urgently needed. This issue has been a hot focus for scientific researchers. Currently, the conventional methods of cancer treatments, i.e., surgery, chemotherapy, and radiotherapy, often lead to side effects and incomplete cancer eradication [3,4]. For instance, chemotherapy involves the use of drugs that are also toxic to normal cells, which could lead to severe side effects in the case of long-term use. Radiotherapy lacks the precision in targeting cancer cells and, thus, can also harm boundary normal cells. Surgery may not always remove the entire cancer and may have the possibility of mistakenly injuring surrounding healthy tissues. To minimize the side effects and enhance therapeutic effectiveness, the development of novel methods is highly needed.

Nanomedicine may be the new promising approach to cancer theranosis (a combined word of diagnosis and therapy). During the last decade, nanomedicine has been the main research focus in cancer theranosis. Researchers have explored various types of nanomaterials for cancer treatments, including gold nanoparticles [5,6,7,8], quantum dots (QDs) [9,10,11,12], upconversion nanoparticles [13,14,15,16], and organic dye-loaded nanoparticles [17,18,19,20] for imaging-guided cancer therapy. However, these materials were often hindered from applications owing to poor water solubility, high toxicity, high costs, and poor photostability. Since the discovery of carbon dots (CDs), they have garnered significant attention for their potential use in cancer theranosis [21,22]. CDs were first identified accidentally by Xu et al. in 2004 in purifying single-walled carbon nanotubes (SWCNTs) [23]. After this, CDs have been extensively studied with a particular focus on developing various synthesis methods, understanding their diverse physicochemical properties, and exploring their applications in various fields [24,25,26,27,28,29].

**Figure 2:**
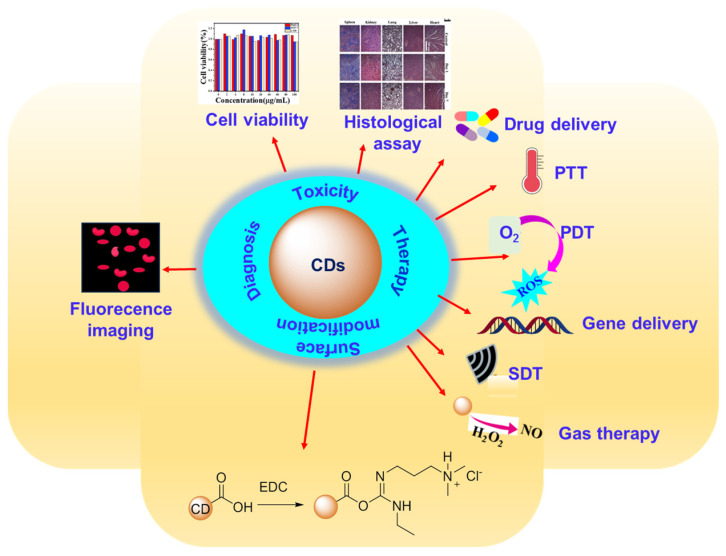
Overview of the main topics of this review: the surface modification, toxicity, and various theranostic application techniques of CDs.

Based on research outcomes up to now, CDs are known for their simple preparation methods, rich hydrophilic surface functional groups, easy surface modifications, high water solubility, stable optical properties, various therapeutic properties (Figure 2), and low toxicity because they are mainly made of nontoxic carbon (C) with additional nontoxic elements such as hydrogen (H) and oxygen (O) [30]. CDs are composed of sp^2^ and sp^3^ carbons and can be made to have hydrophilic functional groups on their surfaces, such as carboxyl (–COOH), amino (–NH_2_), and hydroxyl (–OH) groups [31,32,33]. These rich hydrophilic functional groups of CDs make easy surface modifications to have high loading capacity of various cargoes such as drugs [34,35,36,37,38], photosensitizers [39,40,41,42,43], nucleic acids [44,45,46], and targeting moieties (i.e., transferrin, folic acid, and hyaluronic acid) [47,48,49,50,51,52]. Compared with toxic metal-based QDs [53], CDs are much safer and eco-friendly for biomedical applications as they are free from heavy metals and toxic elements. One of the most notable attributes of CDs is their excellent optical properties, covering absorption and emission from ultraviolet (UV) to near-infrared (NIR) [54], useful for cancer theranosis. These various valuable physicochemical, toxicological, and theranostic properties of CDs make them very promising materials for use in cancer theranosis. The multifunctionality of CDs is useful for cancer theranosis, including fluorescent imaging, drug delivery, photodynamic therapy (PDT), photothermal therapy (PTT), gene therapy, sonodynamic therapy (SDT), and gas therapy, offering new methods for cancer theranosis (Figure 2) [34,40,52,55,56,57,58,59,60,61,62,63,64,65]. Furthermore, the combination of several therapeutic modalities can lead to synergistic effects, further enhancing the therapeutic efficacy.

Despite many review papers on CDs so far [66,67,68,69,70,71,72,73,74,75,76,77,78,79,80,81], there is still a need for an overview focusing on surface functionalization and applications of CDs to cancer theranosis. This review aims to provide an in-depth exploration of the recent developments of CDs applied to cancer theranosis, including imaging (diagnosis) and drug delivery, PTT, PDT, synergistic PTT+PDT, gene delivery, SDT, and gas therapy (therapy), as depicted in Figure 2. In addition, this review discussed the toxicity of CDs both in vitro and in vivo. Finally, we presented the current challenges and future research directions for the clinical translation of CDs.

## 2. Functionalization of CDs

### 2.1. Primary Functionalization

**Figure 3:**
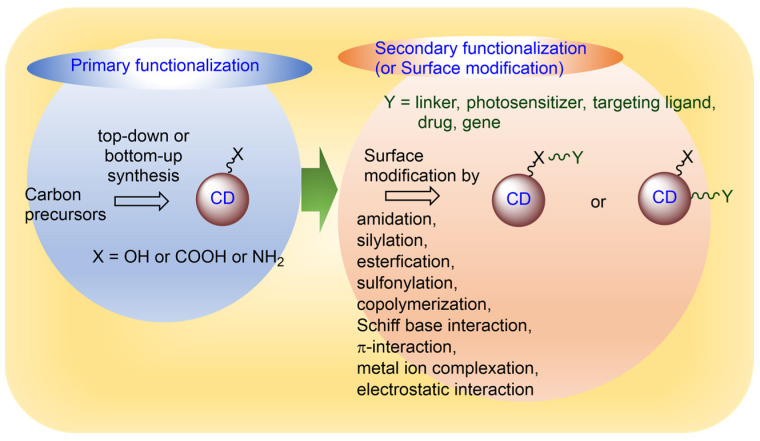
Various functionalizations of CD surfaces.

CDs can be made to possess a variety of hydrophilic functional groups on their surfaces such as carboxyl (–COOH), amino (–NH_2_), and hydroxyl groups (–OH) using proper carbon precursors (i.e., alcohols, acids, and amines) in the bottom-up synthesis or oxidizing CDs obtained from the top-down synthesis (primary functionalization in Figure 3) [82,83]. These functional groups not only enhance the water solubility of CDs but also enable further surface modification through conjugation with various molecules (i.e., linkers, photosensitizers, targeting ligands, anticancer drugs, and genes) (secondary functionalization or surface modification in Figure 3) [84]. The surface modification of CDs with additional molecules plays an important role in tailoring cancer theranosis, while minimizing off-target effects. The various surface-modification methods of CDs are described as follows.

### 2.2. Secondary Functionalization or Surface Modification

As depicted in Figure 3, there exist various surface modification methods, including amidation, silylation, esterification, sulfonylation, copolymerization, Schiff base interactions, π-interactions, metal ion complexation, and electrostatic interactions [84,85,86,87,88].

**Figure 4:**
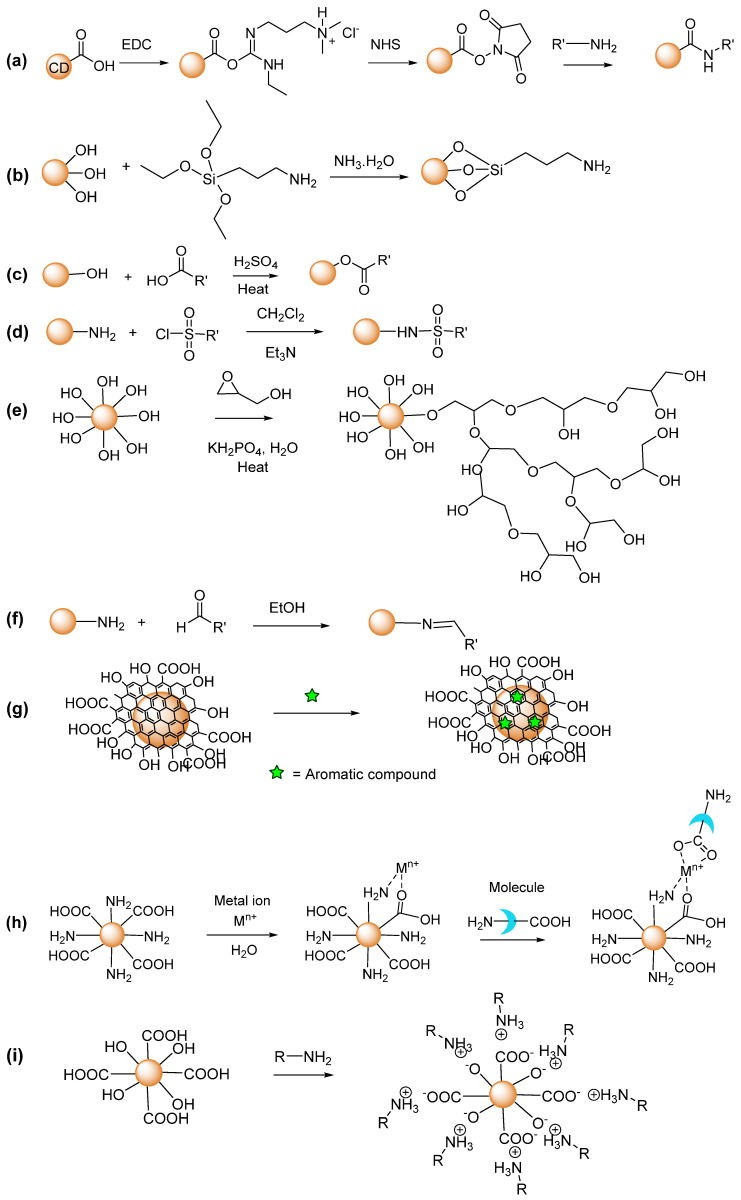
Schematic illustration of surface modifications of CDs via (**a**) amidation, (**b**) silylation, (**c**) esterification, (**d**) sulfonylation, (**e**) copolymerization, (**f**) Schiff-base reaction, (**g**) π-interaction, (**h**) metal ion complexation, and (**i**) electrostatic interaction.

The amidation is widely used to modify the CD surfaces with carboxyl or amine groups in aqueous media (Figure 4a) [89,90]. This method involves the use of coupling reagents such as 1-ethyl-3-(3-dimethylaminopropyl) carbodiimide hydrochloride (EDC) and N-hydroxysuccinimide (NHS). After EDC reacts with carboxyl groups of CDs or molecules, an unstable O-acylisourea intermediate is produced and then stabilized by NHS to form a semi-stable NHS ester, which reacts with amine groups of molecules or CDs to form an amide bond. This modification allows for the conjugation of various molecules, including targeting ligands, photosensitizers, and anticancer drugs [39,43,47,91]. For example, Li et al. prepared CDs with carboxyl groups by oxidizing carbon nanopowder, and covalently conjugated the carboxyl groups of CDs with amine groups of transferrin and doxorubicin to form CD–Trans/Dox using EDC/NHS coupling reaction [91].

The surface modification of CDs via silylation involves the reaction between silane derivatives such as (3-aminopropyl)triethoxysilane (APTES) or tetraethyl orthosilicate (TEOS) and active hydrogens on the CD surfaces in ammonium solution, which results in the covalent O-Si bond (Figure 4b). Compared to TEOS, APTES has the advantage of having amine groups (–NH_2_), which can be used for further functionalization and bonding with carboxyl groups. This functionalization can enhance the biocompatibility and dispersion of CDs in aqueous solution [92]. For example, Niu et al. prepared CDs with hydroxyl groups using alanine and histidine as carbon sources in a hydrothermal method. The as-prepared CDs were then conjugated with APTES through a silylation reaction [92].

The surface modification of CDs via esterification is achieved by reacting alcohol groups of CDs or molecules with carboxylic acids of molecules or CDs to form the ester bond in the presence of heat and an acid catalyst (Figure 4c). This surface modification provides a route for conjugating drugs with –COOH groups with –OH groups of CDs and vice versa. It is known that ester bonds are susceptible to hydrolysis at low pH values, which could contribute to the enhanced drug release at pH 5, making esterification an effective strategy to use in drug delivery systems [93]. For example, Algarra et al. synthesized CDs with hydroxyl groups using lactose as the carbon source. The functionalization of CDs was achieved by adding mercaptosuccinic acid (MSA) through an esterification reaction, resulting in the formation of CDs-MSA [94].

The surface modification of CDs via sulfonylation involves the attachment of sulfonyl groups to the amine groups of CDs. The nucleophilic amine group attacks the sulfonyl chloride to displace the chloride ion to obtain the sulfonated CDs (Figure 4d). This modification greatly increases the water solubility, enabling better dispersion of CDs in aqueous environments. For example, Wang et al. prepared CDs with amine groups using phenylenediamines as the carbon precursor and obtained the 2,4-dinitrobenzene-conjugated CDs (CD-DNS) by reacting 2,4-dinitrobenzenesulfonyl chloride (DNS-Cl) with CDs in the presence of triethylamine and dichloromethane [95].

The surface modification of CDs via copolymerization involves the attachment of cyclic monomers to CDs with hydroxyl groups through polymerization. The ring-opening polymerization is employed in which a cyclic monomer opens up to form a polymer chain that is covalently bonded to the CD surface (Figure 4e). This technique can improve the water dispersibility and biocompatibility of CDs. For example, Li et al. prepared CDs with hydroxyl groups using α-cyclodextrin as the carbon precursor in a hydrothermal treatment and achieved the surface modification of CDs using the anionic ring-opening glycidol polymerization [96]. The copolymerization process resulted in the formation of hyperbranched polyglycerol (HPG) on the surface of CDs. The resulting CDs-g-HPG exhibited strong green fluorescence, low cytotoxicity, and improved hemocompatibility, enhancing their potential as an effective probe for bioimaging.

The surface modification of CDs via Schiff-base reaction involves a primary amine of CDs reacting with carbonyl compounds (aldehyde or ketone), which results in the formation of an imine bond (Figure 4f). Although Schiff bases are stable in alkaline environments, they can undergo hydrolysis into their corresponding amine and carbonyl compounds under acidic conditions, making them suitable candidates for pH-responsive drug delivery systems. Schiff base interactions allow for the attachment of drugs and targeting ligands [97,98]. For example, Liu et al. synthesized CDs with amine groups using a hydrothermal treatment of o-phenylenediamine (oPD) as the carbon precursor [99]. The amine groups of CDs reacted with the aldehyde groups of chlorosalicylaldehyde (CS) to result in the formation of CS-CDs.

The surface modification of CDs via a π-interaction involves the formation of noncovalent bonds between the graphene-like carbon core of CDs and aromatic molecules (Figure 4g). This method provides a noncovalent method for loading hydrophobic drugs onto CDs, improving their delivery, efficacy, and ability to overcome major challenges in chemotherapy, such as drug resistance, short circulation, and short retention in cells [100]. For example, Jiang et al. synthesized APTES-conjugated CDs (Si-CDs) using glycerol and APTES as precursors in a microwave-assisted irradiation [101]. The Si-CDs were further functionalized with dopamine (DA) through the noncovalent π-interaction, resulting in Si-CDs@DA.

The surface modification of CDs via metal ion complexation involves the interaction of metal ions with the amine, hydroxyl, or carboxyl groups of both CDs and chelating molecules through coordination bonds (Figure 4h). This modification can drastically influence the optical and magnetic properties of CDs, making them suitable for multimodal imaging such as fluorescence and magnetic resonance imaging (MRI). This metal–CD complex can also be used as a drug carrier and increase the solubility of the drugs. For example, Chen et al. prepared CDs with amine and carboxyl groups using citric acid and triethylenetetramine as the carbon precursors in a hydrothermal method [102]. Tb^3+^ ions were coordinated with the carboxyl and amine groups of CDs to form CDs-Tb^3+^ complexes, and subsequently, further interacted with guanosine 3′-diphosphate-5′-diphosphate (ppGpp) to form Cd-Tb^3+^-ppGpp complexes.

The surface modification of CDs via electrostatic interaction involves the noncovalent bond formation between the charged CD surfaces and oppositely charged molecules. Here, oppositely charged species are produced owing to proton transfer (i.e., acid-base reaction) between CDs and molecules (Figure 4i). This method is particularly advantageous in applications to gene delivery, where positively charged CDs can form stable complexes with negatively charged DNA or RNA. Similarly, this method can allow for the adsorption of positively charged drugs such as DOX or dopamine onto negatively charged CDs [38]. For example, Jin et al. synthesized CDs with hydroxyl and carboxyl groups using natural carrot as the carbon source in a hydrothermal method [103]. The negatively charged CDs were produced through proton transfer from hydroxyl and carboxyl groups of CDs (acting as acid) to amine groups of polyethyleneimine (PEI) and Nile Blue (NB) chloride (acting as base) to form CDs-PEI/NB nanocomposites through electrostatic interaction.

## 3. In Vitro and In Vivo Toxicity of CDs

CDs are mainly composed of nontoxic C with additional H, O, and N, and their exact compositions depend on carbon precursors. CDs are free from toxic heavy metal ions and thus, promising nontoxic nanoprobes, making them potential candidates for biomedical applications. Ideally, upon administration of CDs for in vivo applications, they should perform their intended functions effectively and subsequently be excreted from the body without causing any adverse effects.

**Figure 5:**
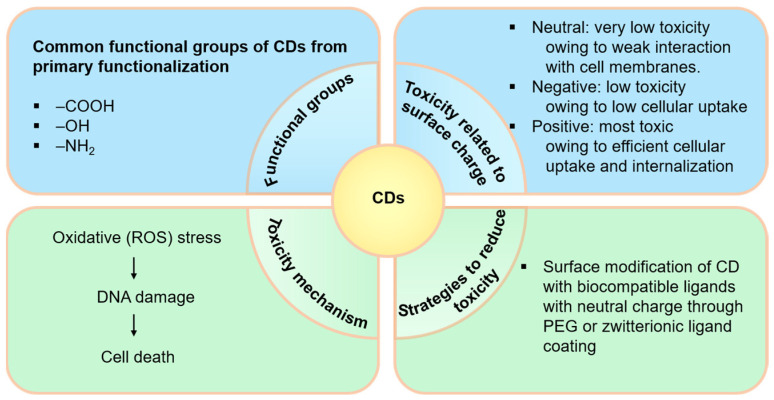
Schematic representation of toxicity related surface conditions of CDs.

Both in vitro and in vivo toxicities of CDs, which are dependent on their chemical composition, size, surface charge, and functionalization, have been widely studied [104,105,106]. As schematically illustrated in Figure 5, for instance, the positively charged CDs (with surface functional groups such as –NH_2_) tend to have higher cytotoxicity compared to negatively charged CDs (with surface functional groups such as –OH and –COOH) or neutral charged CDs (with surface functional groups such as –H) [107]. This can be attributed to a greater capacity of positively charged CDs to interact with negatively charged phospholipids of cell surfaces and their subsequent higher cellular uptake [108]. Furthermore, surface functionalization with biocompatible molecules such as polyethylene glycol (PEG) can significantly enhance their stability and reduce in vivo toxicity [109].

**Figure 6:**
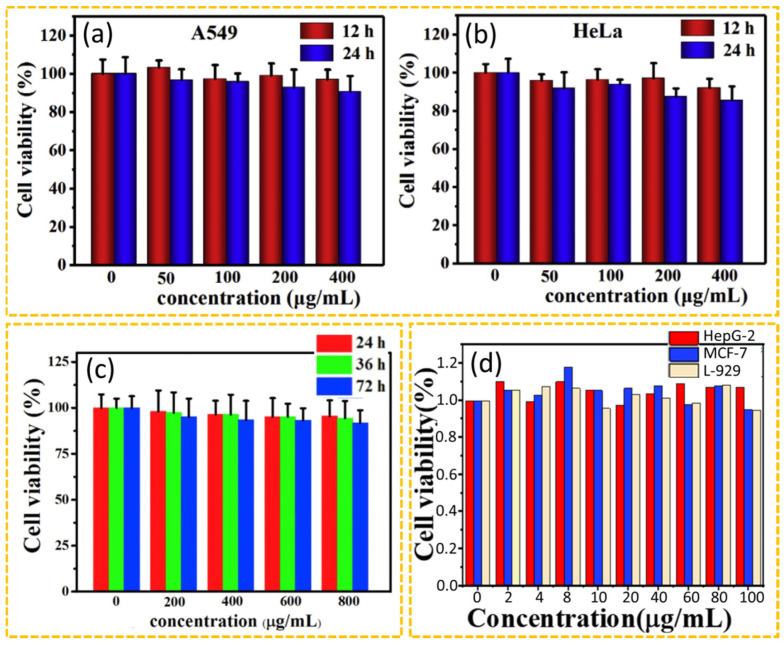
In vitro cell viability of (**a**) A549 and (**b**) HeLa cells after incubation with different concentrations of CDs for 12 and 24 h [113]. (**c**) Cell viability of NIH 3T3 cells after incubation with different concentrations of CDs for 24, 36 and 48 h [114]. (**d**) Cellular cytotoxicity of HepG-2 cells, MCF-7 cells, and L-929 cells after incubation with Green-CDs [115].

The in vitro toxicity is characterized by measuring the cell viability. The common methods include the methylthialazole tetrazolium (MTT), cell counting kit (CCK)-8, and water-soluble tetrazolium (WST)-1 methods [110,111,112]. Until now, numerous studies have demonstrated that CDs have excellent cell viability in vitro. For example, Wang et al. prepared CDs using citric acid, urea, and formamide as carbon sources in a solvothermal method and analyzed the cytotoxicity of CDs using human lung cancer (A549) and Henrietta Lacks (HeLa) cancer cells [113]. Their study revealed that the cell viability remained above 85% even at high CD concentrations (400 μg CDs/mL) after both 12 and 24 h of incubation, demonstrating the low cytotoxicity of CDs (Figure 6a,b). Wang et al. prepared CDs using citric acid and formamide as carbon sources in a solvothermal method and examined the in vitro cytotoxicity of CDs at different CD concentrations (0, 200, 400, 600, and 800 μg CDs/mL) using National Institutes of Health (NIH) 3-day transfer (3T3) cells after 24, 36, and 72 h incubations with CDs (Figure 6c) [114]. Their results demonstrated that the cell viability remained above 90% for all CD concentrations. Guan et al. prepared CDs using gallic acid, carbamide, and PEG400 as carbon sources in a microwave method and demonstrated that CDs exhibited negligible cytotoxicity towards hepatoblastoma (HepG-2), Michigan Cancer Foundation (MCF)-7, and mouse fibroblast (L-929) cell lines at concentrations ranging from 2 to 100 μg CDs/mL over 24 h incubation with CDs (Figure 6d) [115]. Their results exhibited that the cell viability remained approximately 95% for all cells, demonstrating very low cytotoxicity of CDs in vitro.

**Figure 7:**
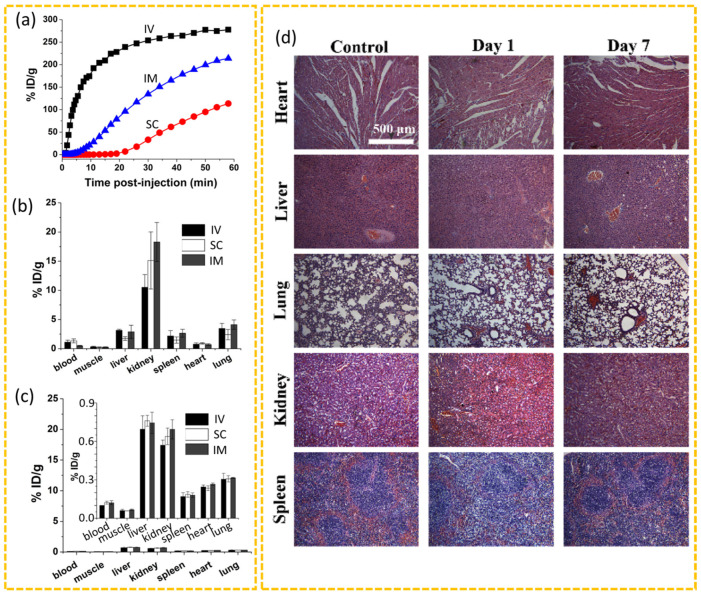
(**a**) Percentages of CDs in urine of injected CDs (% ID/g) as a function of time after IV, IM, and SC administrations. Biodistributions of CDs at (**b**) 1 h and (**c**) 24 h after administration [117]. (**d**) H&E-stained tissue slices (heart, liver, lung, kidney, and spleen) of treated and untreated mice at the 1st and 7th day after IV administration [113].

The in vivo toxicity of CDs using living organisms includes various assays, including biodistribution, clearance, and major organ toxicity (liver, heart, kidney, lung, and spleen) [116]. The biodistribution and clearance of CDs from the body depend on the administration route. Huang et al. synthesized CDs via laser ablation of a carbon target in the presence of water vapor and argon, followed by surface modification with PEG (MW = ~1500 amu). They injected CDs into mice through three different administration routes (2.5 mg CDs/kg), i.e., intravenous (IV), intramuscular (IM), and subcutaneous (SC) administrations, and measured biodistribution and urine clearance of CDs [117]. They observed that the injected CDs could be effectively and rapidly excreted through urine for all three administration routes owing to ultrasmall particle sizes (d = ~3 nm) with the clearance rate of IV > IM > SC (Figure 7a). In addition, their biodistribution study demonstrated that the majority of CDs were accumulated at the kidneys at 1 h after administration in the order IM > IV > SC (Figure 7b), and the remaining CDs in the mice body were very little in all organs at 24 h after administration for all administration routes, indicating that most of the CDs were excreted as urine from the body (Figure 7c). Wang et al. prepared CDs using a simple solvothermal approach and citric acid as the carbon source. They conducted a comprehensive histopathological assay to evaluate the safety and biocompatibility of CDs after sacrificing mice on the first and seventh day after IV administration (administration dose = 25 mg CDs/kg) (Figure 7d) [113]. The analysis involved hematoxylin and eosin (H&E)-stained tissue slices from major organs and revealed no signs of inflammation, necrosis, or other pathological changes up to the seventh day after administration; the normal tissue structures were maintained with well-organized cardiac myocytes, clearly defined liver lobules, intact lung structure, preserved renal glomeruli, and healthy splenic corpuscles. Bao et al. synthesized CDs using citric acid and urea as the carbon precursors in a solvothermal method and also examined histopathological assay using H&E-stained sections of the kidneys, heart, liver, spleen, and lung, and observed no significant signs of organ damage or inflammatory lesions compared to the control groups with no CDs [118]. Sun et al. synthesized CDs through a solvothermal reaction of citric acid and PEI as the carbon precursors and then conjugated chlorin e6 (Ce6) to obtain Ce6-modified CDs. They performed the histopathological assay of CDs using H&E-stained sections of excised major mouse organs (kidneys, heart, liver, spleen, and lungs) and observed no significant inflammation and damage in the mice’s organs compared with those of the control groups, which received phosphate-buffered saline (PBS) [39]. All these in vivo studies demonstrated that CDs are safe and nontoxic. However, almost all toxicity studies on CDs performed so far are acute and single-dose experiments. Therefore, chronic toxicity studies are essential to reveal the potential long-term toxicity of CDs because multiple dosages of CDs are generally required for disease treatments. The CDs exhibited low or negligible toxicity up to 90 days after IV administration (single dose = 20 mg/kg mice) [119]. Mice that received daily doses of 100 mg/kg over 7 days also exhibited low toxicity up to 90 days [120], indicating the low toxicity of CDs. The CDs could adjust the immune function of BALB/c mice by inducing immune Th1 and Tc cell responses, but these effects were not enough to induce the morphological change in immune organs [121], indicating the safety of CDs for the immunological system of mice. However, the mechanism by which CDs modulate the immune system remains unclear. Therefore, to facilitate the commercialization of CDs for clinical applications, more studies are needed, and it will be valuable to develop standard toxicity evaluation protocols for acute and chronic (>90 days) toxicity and immunological assays.

## 4. Applications of CDs in Cancer Theranosis

Recently, the application of CDs in cancer theranosis has emerged as a promising and effective method [122,123]. CDs can be ideal systems for cancer theranosis because of their diagnostic (i.e., imaging) and therapeutic (i.e., drug delivery, PTT, PDT, gene delivery, SDT, and gas therapy) functions. In addition, as provided in Table 1, compared to micelles, liposomes, nanogels, and nanoparticles, CDs can offer several advantages, including stable photoluminescence, low cytotoxicity, and easy synthesis and surface modification, making them a preferred choice for cancer theranostic applications [124].

### 4.1. Cancer Diagnosis via Fluorescence Imaging

#### 4.1.1. In Vitro Diagnosis

Due to their unique optical properties covering UV to NIR, CDs are highly suitable for various imaging applications for cancer diagnosis [134,135,136,137,138]. This coverage results from several origins. One is the particle size of CDs; by increasing the particle size, the emission wavelength increases [139]. In addition, the graphitic-N, -C-N, -C-O, and -COOH groups [140] and the heteroatom doping, such as N, S, P, or B [141], can change the emission wavelength. Therefore, the wavelength tuning is related to both core synthesis and surface modification and depends on carbon precursors, synthesis conditions, and synthesis methods. Their NIR fluorescence (wavelength = 700–900 nm) with tissue penetration depth of 5–7 mm, depending wavelength and tissue type, is particularly useful as it mitigates interference from background fluorescence produced by tissues within the body, thereby enhancing the signal-to-noise ratio and also allowing a deep tissue imaging; for example, Fathi et al. reported the penetration depth of 8.5 mm in the NIR imaging [142]. Zheng et al. demonstrated a remarkable tissue penetration depth of 20 mm in chicken breast tissue [143]. Unlike traditional dyes and fluorophores, CDs can offer tunable wavelength properties, making them more attractive for cancer diagnosis [144]. The discrimination of cancer cells from normal cells is quite challenging in cancer diagnosis [145,146,147]. CDs can be designed to selectively bind to specific cancer cells by conjugating them with cancer-specific ligands, such as folate receptors overexpressed in cancer cells, and used to distinguish cancer cells from normal cells through fluorescent imaging [148,149,150].

**Figure 8:**
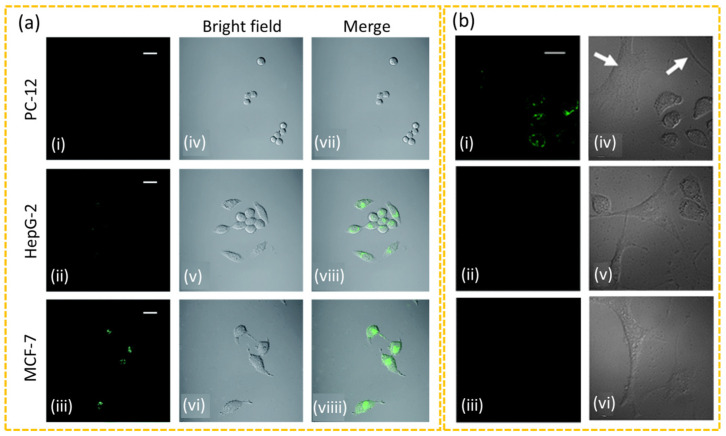
(**a**) Confocal laser scanning microscope images of three different cancer cells after incubation with FA-CDs for 4 h at 37 °C (1 mg CDs/mL). The fluorescence images of (**i**) PC-12, (**ii**) HepG-2, and (**iii**) MCF-7 cells incubated with FA-CDs. The bright field images of (**iv**) PC-12, (**v**) HepG-2, and (**vi**) MCF-7 cells and the overlay images of (**vii**) PC-12, (**viii**) HepG-2, and (**viiii**) MCF-7 cells. Scale bar, 20 μm [151]. (**b**) Confocal laser scanning microscope images of the cell mixture of NIH-3T3 and HeLa cells after incubation (**i**) with and (**ii**) without (as control) FA-CDs (50 μg CDs/mL) for 6 h at 37 °C and (**iii**) NIH-3T3 cells incubated with FA-CDs (as another control). (**iv**–**vi**) The differential interference contrast images of the corresponding cells on the right panels: in the image (**iv**), the arrows indicate NIH-3T3 cells. Scale bar, 20 μm [152].

Zhao et al. synthesized folic acid (FA)-conjugated fluorescent CDs (FA-CDs) for targeted imaging of the Michigan Cancer Foundation (MCF)-7 human breast cancer cells, human liver cancer (HepG2) cells, and pheochromocytoma (PC)-12 rat cancer cells using confocal laser scanning microscopy [151]. They demonstrated that the expression levels of folate receptors in cancer cells depended on cancer cell types; the expression levels of folate receptors were MCF-7 > HepG-2 > PC-12 cells (Figure 8a(i–viiii)). Song et al. developed FA-CDs to distinguish cancer cells from normal cells by culturing and analyzing a mixture of NIH-3T3 normal and HeLa cancer cells [152]. They observed that after 6 h incubation of the cell mixture with FA-CDs, only the HeLa cells exhibited bright fluorescence, while NIH-3T3 cells did not (Figure 8b(i–vi)), demonstrating the selective targeting of the FA-CDs to cancer cells.

#### 4.1.2. In Vivo Diagnosis

**Figure 9:**
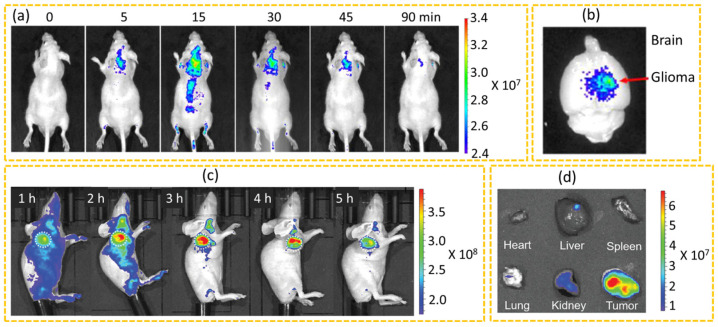
(**a**) In vivo imaging of C6 glioma-bearing mice after tail vein administration of CD-Asp and (**b**) ex vivo imaging of the brain 90 min after IV administration of CD-Asp [134]. (**c**) In vivo imaging of cancer-bearing mice after administration with LAAM GUA-CDs/Cy5-siBcl-2 via tail vein and (**d**) ex vivo fluorescence images of cancers and organs after dissection at 5 h after IV administration [153]. The color scale indicates intensities in units of radiance.

CDs are becoming widely used for in vivo cancer imaging owing to their low toxicity and high quantum yields, showing promising results in cancer diagnosis. The in vivo cancer imaging is typically performed using mice or zebrafish models to examine the biodistribution, clearance, and uptake of CDs in cancer imaging and treatment. For in vivo imaging, longer excitation wavelengths are preferred as they can penetrate tissues more effectively, facilitating deep tissue imaging. However, most CDs reported so far are those emitting in the blue to green region. Zheng et al. used D-glucose and L-aspartic acid (Asp) as carbon sources in pyrolysis to synthesize CD-Asp, which exhibited targeting capability towards C6 brain cancer glioma without conjugation of any additional cancer-targeting ligands [134]. In vivo fluorescence imaging showed rapid accumulation of CD-Asp in the brain 5 min after tail vein administration, with the highest contrast of CD-Asp 15 min after administration (Figure 9a). Furthermore, the ex vivo imaging of the brain confirmed that the CD-Asp targeted the glioma site, showing great potential for the diagnosis of brain cancer cells (Figure 9b). Xu et al. developed large amino acid-mimicking CDs via guanidinium modification (LAAM GUA-CDs) to efficiently load small interfering or silencing RNA (siRNA) to target cancer cells in vivo [153]. In vivo cancer targeting of the LAAM GUA-CDs/Cy5-siRNA was found to accumulate within 1 h and peak at 3 h after IV administration (Figure 9c), while free Cy5-siRNA showed minimal cancer localization due to rapid enzymatic degradation during circulation. Furthermore, the biodistribution analysis revealed that the administration of free Cy5-siRNA resulted in no signal at the cancer and weak fluorescence signals in major organs with the exception of the kidneys, indicating a short circulation time due to renal excretion of the free Cy5-siRNA whereas the LAAM GUA-CDs/Cy5-siRNA demonstrated significantly stronger fluorescence signals at the cancer site compared with other organs owing to their active cancer targeting (Figure 9d). Su et al. reported red-emissive CDs that could penetrate the nuclei of both cancer cells and cancer stem cells [154]. The DOX was loaded onto the surface of the CDs at a concentration of 30 µg/mL. The bioimaging and biodistribution of DOX-loaded CDs (CSCNP-R-CDs/DOX) were evaluated in vivo after IV administration into the cancer-bearing Balb/c nude mice. At 6 h after administration, weak fluorescence at the cancer site indicated CSCNP-R-CQDs/DOX accumulation and reached its peak at 12 h after administration, confirming the effective targeting of the CDs at the cancer site. Ex vivo fluorescence imaging of isolated organs, including the cancer, liver, kidney, lung, and brain, further revealed that the cancer site exhibited the strongest fluorescence intensity at 12 h after administration, indicating that the CSCNP-R-CQDs/DOX were mainly concentrated at the cancer.

### 4.2. Cancer Therapy via Drug Delivery, PTT, PDT, PTT+PDT, SiRNA Delivery, SDT, and Gas Therapy

#### 4.2.1. Drug Delivery

CDs have emerged as promising nanocarriers for targeted drug delivery. By surface functionalization or surface modification of CDs, they can be engineered to transport therapeutic drugs to specific target sites. The fluorescence properties of CDs allow for the visualization of the drug delivery to the cancer site. The DOX is commonly used in chemotherapy because of its effective anticancer activity. However, its use is limited owing to its nonspecific nature, poor water solubility, and toxicity to normal cells [155]. Thus, the delivery of DOX using CDs can be an effective approach to improve the therapeutic effects and minimize the side effects of DOX through enhanced water solubility and specificity.

**Figure 10:**
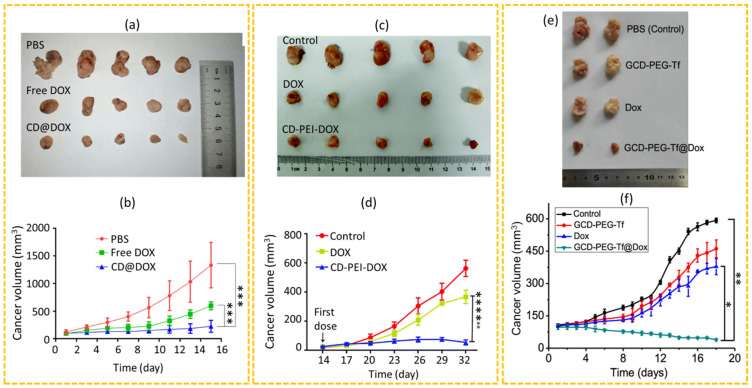
(**a**) Photographs of dissected cancers on the 15th day after IV administration and (**b**) cancer volume changes after IV administration into 4T1 cancer-bearing mice (*** *p* < 0.001) [156]. (**c**) Photographs of dissected cancers on the 33rd day after IV administration into MHCC-97L cancer-bearing mice and (**d**) cancer volume changes after administration (**** *p* < 0.0001) [157]. (**e**) Photographs of the dissected cancers on the 18th day after IV administration and (**f**) cancer size changes after IV administration (* *p* < 0.01; ** *p* < 0.001) [158]. *p*-values were calculated by Student’s *t*-test.

Li et al. synthesized hyaluronic acid-modified CDs (HA-CDs) for targeted drug delivery using breast cancer (4T1)-bearing BALB/c mice [156]. HA was used as a targeting ligand of cluster determinant 44 receptors overexpressed on cancer cells. The DOX was loaded onto HA-CDs through 4-carboxybenzaladehyde (p-CBA) as a pH-sensitive linker, enabling controlled drug release under acidic conditions at the cancer site. The enhanced cancer accumulation of HA-CD@p-CBA-DOX could be attributed to the active targeting by HA and the passive targeting by the EPR effect. The study demonstrated that the HA-CD@p-CBA-DOX significantly inhibited cancer growth, showing a cancer growth inhibitory rate of 78.53%, compared with 57.07% of the free DOX (Figure 10a,b). Hailing et al. investigated the anticancer efficacy of CD-PEI-DOX in BALB/c nude mice bearing liver cancers (MHCC-97L) [157]. In their study, the cancer-bearing mice were injected with PBS, free DOX, and CD-PEI-DOX through the tail veins. The results demonstrated that all DOX treatment groups exhibited significant inhibition of cancer growth compared to the PBS control group (Figure 10c,d), but the CD-PEI-DOX group exhibited the lowest cancer growth rate compared to the other groups. These findings suggested that the CD-PEI-DOX could potentially improve the therapeutic efficacy of DOX by maximizing its accumulation at the cancer site. Li et al. developed a targeted drug delivery system using green fluorescent CDs (GCDs) conjugated with PEG and transferrin (Tf) to load DOX for the effective treatment of cancer [158]. In their work, cancer-bearing mice were divided into four groups with IV administration: control (PBS), GCD–PEG–Tf, free DOX, and GCD–PEG–Tf@DOX. The results showed that both the GCD–PEG–Tf and free DOX groups exhibited rapid cancer growth, as shown in Figure 10e,f, whereas the GCD–PEG–Tf@DOX-treated mice exhibited significant cancer growth inhibition. This enhanced cancer suppression was attributed to the ability of GCD–PEG–Tf@DOX to selectively target cancer cells and longer circulation time in vivo, highlighting its potential as a targeted drug delivery and cancer therapy.

#### 4.2.2. PTT

The PTT has emerged as a promising approach for cancer treatments due to its high therapeutic efficacy and low side effects [159,160]. The PTT utilizes light-absorbing photothermal agents (PTAs), which absorb NIR light and convert it into heat [159]. The elevated heat induces cancer cell death, as cancer cells are more heat-sensitive than normal cells. The mechanism of PTT involves the delivery of PTAs to the cancer site. Upon NIR irradiation, the PTAs generate localized heat, leading to the denaturation of proteins, disruption of cancer cell membranes, and subsequent thermal ablation of cancer cells. Among the various PTAs, CDs have shown great potential due to their strong NIR absorption and high photothermal conversion efficiency (PCE). CDs possess π-conjugated electrons in the sp^2^ domains, which enable efficient absorption and transformation of NIR light into heat [161]. In addition, heteroatom (N or S)-doped CDs exhibited enhanced NIR absorption and higher PCE, making them highly effective PTAs for cancer therapy.

**Figure 11:**
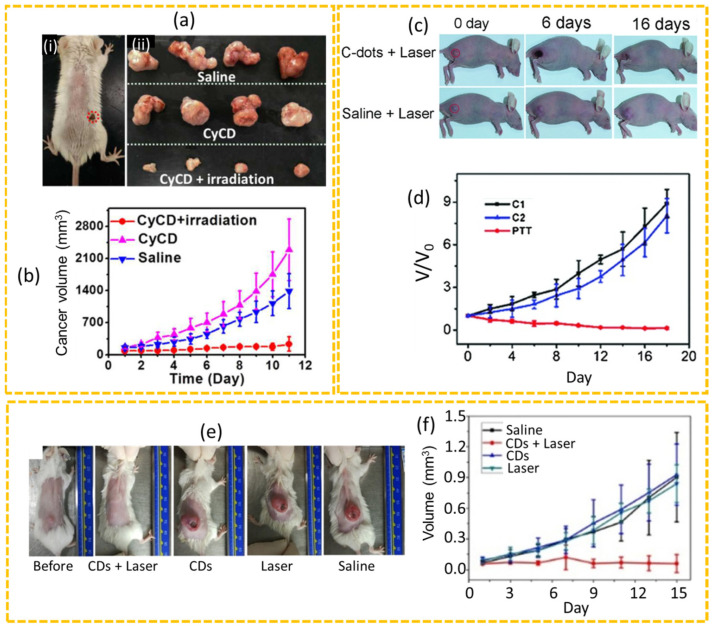
(**a**) (**i**) Photograph of CT26 cancer-bearing mouse: cancer is labeled with a red dotted circle after PTT and (**ii**) photographs of the excised cancers on the 11th day after treatment. (**b**) Cancer volume growth of saline, CyCD, and CyCD + irradiation groups after treatment [162]. (**c**) Photographs of the cancer-bearing mice on different days after treatment and (**d**) relative cancer volume (V/V_0_) growth after treatment; C1 = CDs only, C2 = saline + laser, and PTT = CDs + laser (V: cancer volume and V_0_: initial cancer volume before PTT) [163]. (**e**) Photographs of the cancer-bearing mice on the 15th day after PTT, CD, laser, or saline treatments and (**f**) cancer volume change after treatment [164].

For example, Zheng et al. used cyanine dye (CyOH) and poly(ethylene glycol) (PEG800) as the carbon sources to synthesize CDs (CyCDs) in a solvothermal method and investigated the in vivo therapeutic effects of multifunctional CDs (CyCD) using CT26 cancer-bearing BALB/c mice [162]. These CDs exhibited NIR absorption and emission in the range of 600–900 nm with a high PCE of 38.7%. The incorporation of PEG800 enhanced the hydrophilicity and photostability of the CDs, making them ideal candidates for cancer-targeted imaging and PTT. The treatment involved intracancerously injected CyCDs at a dosage of 4.0 mg CyCDs/kg body weight, followed by exposure to an 808 nm laser for 5 min to maintain the cancer temperature of approximately 45 °C. The treatment was administered four times at a two-day interval. After 11 days, the mice were sacrificed, and cancer weights were measured. The results indicated a significant cancer inhibitory rate of 91% in the CyCD + irradiation group as shown in Figure 11a(i,ii),b, whereas cancers in the saline and CyCD only groups exhibited a rapid growth with cancer volumes increased by 8- and 13-fold the initial volumes, respectively, demonstrating the potential of CyCDs to serve as a promising candidate in cancer theranosis. Ge et al. investigated the PTT of HeLa cancer-bearing nude mice using CDs that were synthesized using polythiophene phenylpropionic acid via the hydrothermal method [163]. The treatment group received CDs via IV administration, followed by irradiation with a 671 nm laser for 10 min, while one control group was treated with CDs only without laser irradiation (labeled as C1), and the other control group received saline with laser treatment (labeled as C2) (Figure 11c,d). Results indicated that cancer tissues of the two control groups (C1 and C2) continued to grow without any significant therapeutic effect, whereas the mice that received both CDs and laser irradiation exhibited substantial empyrosis at the cancer sites, leading to a significant suppression of cancer growth. Moreover, the dark burn scab that formed on the skin of the mice was detached on day 16 after treatment, showing a complete recovery and demonstrating that CDs should be a prominent candidate for cancer theranosis. Zhao et al. investigated the PTT of CDs using 4T1 subcutaneous xenograft murine models [164]. These CDs exhibited broad absorption with a high PCE of 54.7% under 808 nm laser irradiation for 10 min. The study revealed that the in vivo PTT performance of the CDs was significantly effective in inhibiting cancer growth compared to control groups; neither the CDs nor the laser treatment alone effectively destroyed cancer cells or reduced cancer growth (Figure 11e,f).

#### 4.2.3. PDT

The PDT is a non-invasive cancer treatment method that utilizes photosensitizers, light, and oxygen to generate ROS, which induces cancer cell apoptosis and necrosis. The PDT relies on the ability of photosensitizers to absorb light and transfer energy to nearby oxygen molecules, producing cytotoxic ROS such as singlet oxygen (^1^O_2_) and free radicals. In recent years, CDs have emerged as promising candidates for PDT due to their strong light absorption in the UV to NIR regions. CDs can serve as self-photosensitizers, which transfer energy to surrounding oxygen molecules [165,166,167]. Therefore, CDs alone can be used for PDT, but are less effective compared with conventional photosensitizers such as porphyrin. To enhance the effectiveness of PDT, CDs can be combined with traditional photosensitizers such as porphyrin [168] and chlorin derivatives [39]. Although traditional photosensitizers are more powerful for PDT than CDs, their poor water solubility limits their PDT applications. For example, CDs conjugated with Ce6 highly enhanced the ROS production compared with Ce6 alone. In addition, CDs can be functionalized with targeting molecules such as FA to increase the selectivity for cancer cells, further improving the PDT efficiency [169,170].

**Figure 12:**
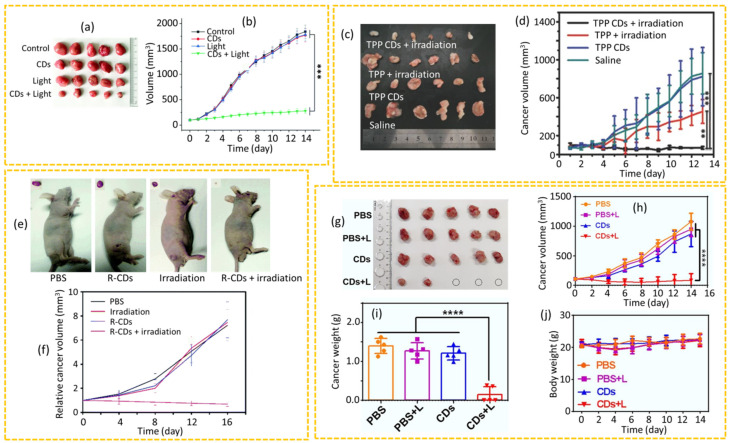
(**a**) Photographs of excised cancers after two weeks of PDT and (**b**) cancer volume changes in 4T1-bearing mice over time after PDT (*** *p* < 0.001) [171]. (**c**) Photographs of excised cancers on the 13th day after PDT and (**d**) cancer volume changes in H22-bearing mice over time after PDT (** *p* < 0.01; *** *p* < 0.001) [168]. (**e**) Photographs of mice and excised cancers from the sacrificed mice on the 16th day after PDT and (**f**) cancer volume changes in A549-bearing mice over time after PDT [165]. (**g**) Photographs of excised cancers on the 15th day after PDT, (**h**) cancer volume changes in CT26-bearing mice over time after PDT, (**i**) cancer weights on the 15th day after PDT, and (**j**) body weight changes in CT26-bearing mice over time after PDT (**** *p* < 0.0001) [59]. *p*-values were calculated by Student’s *t*-test.

Yue et al. prepared CDs using riboflavin as the carbon source and ethylenediamine as a cross-linking agent in a hydrothermal method and used 4T1-bearing mice to evaluate the effectiveness of PDT [171]. As shown in Figure 12a,b, the cancer volumes in the PDT-treated group (CDs + 365 nm UV light for 10 min) did not exhibit significant cancer growth over time, whereas the cancer volumes in the other treatment groups receiving either CDs only or light only, exhibited a rapid cancer growth similar to that of the control group. After two weeks of treatment, the PDT effectively inhibited the cancer growth, but did not reduce the cancer size due to the limited penetration depth of UV light. As a result, the authors suggested that this method will be more suitable for treating superficial skin cancers or inflammatory wounds. In a study by Li et al., porphyrin-based CDs (TPP CDs) were prepared using mono-hydroxylphenyl triphenylporphyrin (TPP) and chitosan in a hydrothermal method and used for PDT of hepatocarcinoma 22 (H22) xenograft models [168]. The TPP CDs exhibited excellent photostability, good water solubility, good biocompatibility, and efficient ROS generation upon 625 nm irradiation for 1 h. The results indicated a significant reduction in cancer size from 100 mm^3^ to 56 mm^3^ in the PDT group, while the saline control and TPP CD-only groups exhibited dramatic increases in cancer size to approximately 800 mm^3^ (Figure 12c,d). Notably, TPP CDs were able to inhibit cancer growth more effectively (82.87%) than TPP (52.74%) owing to the poor water solubility of TPP. The incorporation of stable heteroatoms such as nitrogen or phosphorus into the CD structure has been shown to promote ROS generation [111]. For example, Zhao et al. synthesized nitrogen and phosphorus-co-doped red-emissive CDs (R-CDs) using o-phenylenediamine and phosphoric acid as the raw materials in a hydrothermal method, which enhanced the ROS production of ^1^O_2_ [165]. These R-CDs were utilized as photosensitizers in the PDT of human lung (A549) cancer-bearing nude mice under 532 nm laser irradiation for 4 min. The PDT with R-CDs significantly inhibited cancer growth, whereas no significant differences in cancer volume were found among the three control groups (PBS, 532 nm laser irradiation, and R-CDs), confirming the efficacy of R-CDs as photosensitizers for PDT of cancer (Figure 12e,f). Recently, the use of afterglow imaging with CDs in imaging-guided PDT has represented a significant advancement for cancer therapy. Li et al. investigated the anticancer efficacy of CDs against a subcutaneous xenograft cancer model of mouse colon carcinoma (CT26) cells implanted in the armpit of Balb/c mice using CDs synthesized in a solvothermal method by heating the mixture of Ce6 and PEI in N,N-dimethylformamide solution at 160 °C for 2 h [59]. These CDs demonstrated deep tissue penetration, reaching depths of up to 10 mm in chicken breast tissue. After 7 days of cancer growth, the mice were divided into four treatment groups: PBS, PBS + L, CDs, and CDs + L, where “L” refers to 685 nm laser treatment for 10 min. The results indicated that the cancer sizes in the PBS, PBS + L, and CDs groups exhibited significant cancer growth, suggesting that neither CDs nor laser irradiation alone was effective in inhibiting cancer growth, as shown in Figure 12g,h, whereas the CDs + L group exhibited a drastic reduction in cancer growth. After 15 days of the treatment, the mice were sacrificed, and analysis revealed that the cancer weight in the CDs + L group was the smallest among all groups (Figure 12i), with three cancers completely ablated among five cancers (Figure 12g). Furthermore, the study demonstrated the negligible side effects of CDs, as evidenced by the negligible mouse weight changes for all groups (Figure 12j).

#### 4.2.4. Synergistic PDT+PTT

As discussed above, CDs can be used for both PDT and PTT, providing multiple choices for therapeutic applications. Recently, synergistic PTT and PDT using CDs have shown great promise in improving monotherapy mode by simultaneously generating heat and ROS [39,172,173,174,175,176].

**Figure 13:**
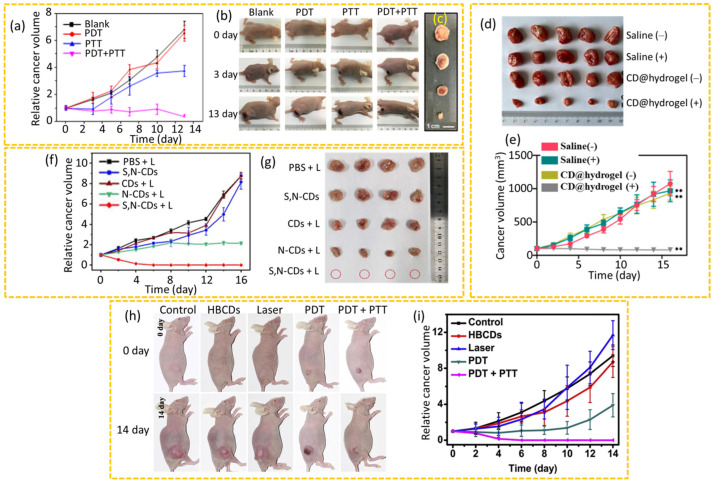
(**a**) Cancer volume growth curves over time of the four 4T1 cancer-bearing mice groups after treatments, (**b**) photographs of mice on the 0th, 3rd, and 13th days after treatments and (**c**) excised cancers on the 13th day after treatments [39]. (**d**) Photographs of excised cancers from four 4T1 cancer-bearing mice groups on the 16th day after treatments and (**e**) cancer volume growth curves over time (** *p* < 0.01) (–: no irradiation and +: irradiation) [177]. (**f**) Cancer volume changes in HeLa cancer-bearing mice over time and (**g**) photographs of excised cancers on the 16th day after treatments (L: laser) [179]. (**h**) Photographs of 4T1 cancer-bearing nude mice on the 0th and 14th days after treatments and (**i**) cancer volume growth curves over time [176]. The relative cancer volume is the cancer volume with respect to initial cancer volume before therapeutic treatment. *p*-values were calculated by Student’s *t*-test.

In a study by Sun et al., a small amount of photosensitizer Ce6 was anchored onto CDs to enhance the efficacy of cancer phototherapy with a relatively low laser irradiation power [39]. They demonstrated the superior efficacy of the synergistic PDT+PTT mode compared to either single mode of PDT and PTT under a relatively low-power laser irradiation. The in vivo assessments of the PDT and PTT efficacy of Ce6-modified red emissive CDs (Ce6-RCDs) were performed using 4T1 cancer-bearing nude mice. The mice were separated into four treatment groups: a blank group (PBS + laser), a PDT group (free Ce6 + laser), a PTT group (RCDs + laser), and a combined PDT + PTT group (Ce6-RCDs + laser), in which a 671 nm laser was irradiated for 10 min. The results revealed that the PDT group exhibited negligible cancer growth inhibition, with cancer volumes increasing to about 6.5 times their initial size after 13 days of treatment, similar to the blank group (Figure 13a–c). The PTT group showed some cancer growth inhibition, but the cancer volume still increased to approximately 3.5 times the initial size. In contrast, the PDT + PTT group demonstrated a significant cancer growth suppression, with cancer volumes shrinking to around 30% of their initial size after 13 days of treatment and also exhibited signs of cancer necrosis and black scars at the cancer site just three days after treatment (Figure 13b,c), whereas the blank and PDT groups showed a rapid cancer growth three days after treatment. Recently, an injectable CD-based hydrogel was developed for simultaneous PTT and PDT [175,177,178]. Yue et al. prepared a novel injectable hydrogel using CDs and HA (CD@ Hydrogel) for PTT and PDT of cancer in which HA was used as a hydrogel precursor [177]. The anticancer activity of CD@Hydrogel in vivo was evaluated using 4T1 cancer-bearing mice. As shown in Figure 13d,e, the CD@Hydrogel + NIR (660 nm for 600 s) group exhibited the most pronounced inhibitory effect on cancer growth, with no significant inhibitory effect in the other three groups. In addition, there were no significant changes in body weight for all groups, offering a simple and efficient strategy for cancer phototherapy. Bai et al. evaluated the anticancer efficacy of sulfur (S) and nitrogen (N)-codoped CDs, N-doped CDs, and CDs using HeLa cancer-bearing mice [179]. As illustrated in Figure 13f,g, the PBS + laser (660 nm and 5 min irradiation), S and N-CDs, and CDs + laser groups showed no significant anticancer effects, and the N-CDs + laser group exhibited limited cancer inhibition. However, the S and N-CDs + laser group exhibited almost complete ablation of cancer. Jia et al. synthesized hypocrella bambusae (HB)-derived R-CDs (HBCDs) for imaging-based PTT and PDT (635 nm and 10 min laser irradiation) [176]. The study involved five groups of 4T1 cancer-bearing nude mice. As shown in Figure 13h,i, while the PDT group showed some cancer reduction, it was not sufficient for complete elimination of the cancer, whereas the synergistic PDT+PTT group achieved total cancer inhibition without recurrence at the 14th day after treatment, indicating superior efficacy. The control groups, HBCDs, and laser irradiation alone exhibited rapid cancer growth, showing no therapeutic effect. Importantly, the body weight of the mice remained the same throughout the treatments, confirming the low biotoxicity of HBCDs in vivo.

#### 4.2.5. siRNA Therapy

The siRNA therapy is a promising alternative for the treatment of cancer [180,181,182]. It lies in the use of effective carriers of siRNA into cancer cells. One valuable strategy emerging in this field is the use of CDs as siRNA carriers, which are small in size with unique optical properties, good water solubility, and good biocompatibility. The charge interactions between CDs and siRNA are the key to successful siRNA transfer. The siRNA possesses a negative charge due to the presence of phosphate groups in its backbone structure, making it necessary for CDs to possess a positive charge through surface modifications with positively charged molecules such as PEI. In addition, the fluorescence properties of CDs facilitate tracking of their delivery. Several studies have demonstrated the potential use of CDs in siRNA delivery and therapy [46,61,153,183,184,185,186,187,188,189].

**Figure 14:**
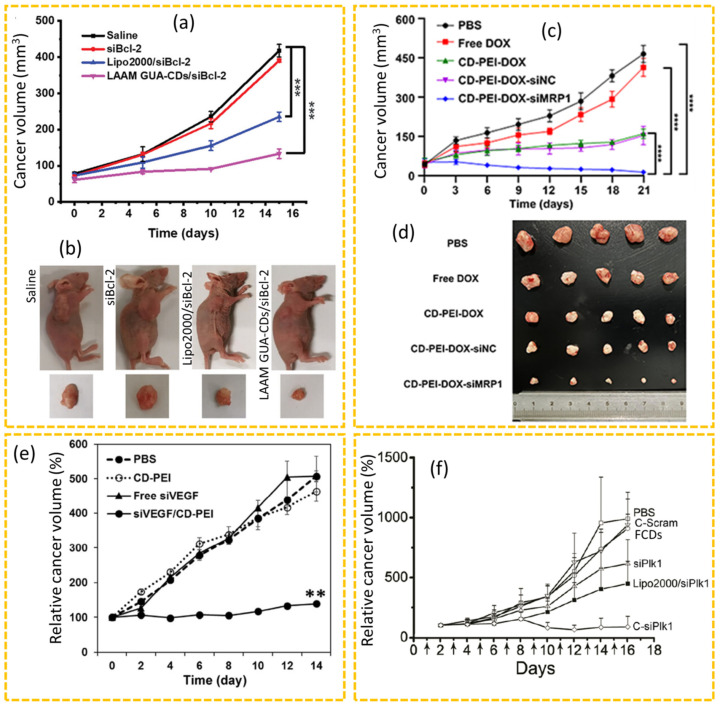
(**a**) Cancer volume growth curves over time (*** *p* < 0.001) and (**b**) photographs of HeLa cancer-bearing mice and excised cancers on the 15th day after treatment [153]. (**c**) Cancer volume growth curves of A549/ADM cancer-bearing subcutaneous xenograft model mice over time (**** *p* < 0.0001) and (**d**) photographs of excised cancers on the 21th day after treatment [188]. (**e**) Relative cancer volume changes over time of the MDA-MB-231 cancer-bearing mice (** *p* < 0.01) [185]. (**f**) Cancer volume changes over time of A375 cancer-bearing mice (The arrows on the axis represent the days of drug administration) [183]. *p*-values were calculated by Student’s *t*-test.

Xu et al. performed targeted siRNA delivery using LAAM GUA-CDs for cancer therapy of HeLa cancer-bearing mice in which the LAAM GUA-CDs enabled efficient siRNA loading and targeted delivery to the cancer site [153]. The Bcl-2 siRNA (siBcl-2) can down-regulate the antiapoptotic protein Bcl-2 expression in cancer cells, which is responsible for inhibiting cancer cell growth. They demonstrated that LAAM GUA-CDs/siBcl-2 achieved a 78% gene silencing efficiency in vitro and a cancer inhibition efficiency of 68% in vivo, as shown in Figure 14a and Figure 14b, respectively. Notably, the transfection efficiency of LAAM GUA-CDs/siBcl-2 was twice as high as that of transfection reagent Lipofectamine2000 conjugates with siBcl-2 (Lipo2000/siBcl-2), making LAAM GUA-CDs/siBcl-2 a promising tool for gene cancer therapy. Yu et al. performed a study to evaluate the efficacy of the DOX and siRNA co-delivery system (i.e., CD-PEI-DOX-siMRP1) in inhibiting cancer growth using lung cancer xenograft models in nude mice [188]. The siRNA targeted the multidrug-resistant proteins MRP1 in lung cancer to down-regulate them and consequently, enhance the chemotherapy efficiency of DOX. The CD-PEI-DOX-siMRP1 exhibited a considerably stronger signal in the cancer site than the DOX group, owing to its enhanced cancer targeting. As shown in Figure 14c,d, the cancers treated with CD-PEI-DOX-siMRP1 were considerably smaller than those of the other treatment groups, demonstrating that the co-delivery system was superior to the other treatments in effectively inhibiting cancer growth. Kim et al. developed an efficient siRNA delivery system using CD-PEI to effectively knock down the vascular endothelial growth factor (VEGF) gene expression in vitro and in vivo [185]. They investigated the anticancer efficacy of the siRNA/CD-PEI in a human breast cancer xenograft mouse model through IV administration of siVEGF/CD-PEI. The results indicated a significant inhibition of cancer growth in the siVEGF/CD-PEI-treated group, as illustrated in Figure 14e, whereas the groups treated with CD-PEI and free siVEGF did not exhibit any significant changes in cancer size compared to the control group. The authors demonstrated that the CD-PEI not only facilitated effective siRNA delivery but also protected the siRNA from degradation, thereby enhancing its therapeutic efficacy in vivo. Zhang et al. developed fluorescent CD (FCD)-based Plk1 targeting siRNA (siPlk1) (C-siPlk1) to perform in vivo cancer inhibition experiments using Balb/c nude mice implanted with human melanoma cancer (A375) cells [183]. The C-siPlk1 down-regulated the expression of polo-like kinase-1 (Plk1), a master regulator of mitosis. After one week of the treatment, C-siPlk1 demonstrated significant efficacy in inhibiting cancer growth compared to the other groups (Figure 14f), in which the cancer volumes in the PBS-only, FCDs-only, and FCDs/scrambled siRNA (C-Scram) groups increased sharply. In addition, the administration of free siPlk1 and Lipo2000/siPlk1 resulted in at least a four-fold increase in cancer volume compared to the pre-treatment levels.

#### 4.2.6. SDT

**Figure 15:**
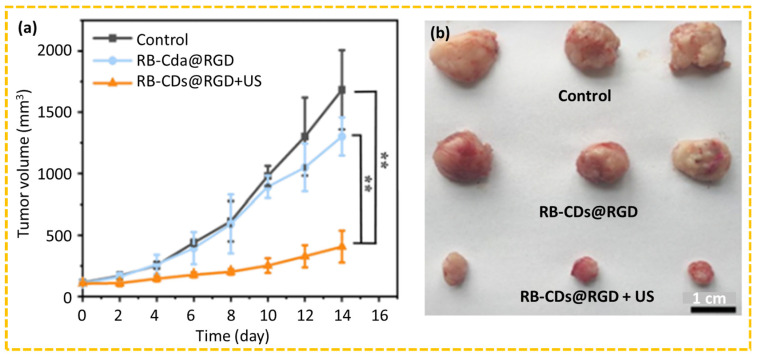
In vivo evaluation of anti-tumor performance of RB-CDs@RGD in C6 tumor-bearing nude mice using SDT: (**a**) tumor volume growth curves up to 14 days and (**b**) photographs of excised tumor tissues after 14 days. Student’s *t*-test (** *p* < 0.01). *p* < 0.05 is considered to be statistically significant [192].

The SDT is a non-invasive therapeutic modality that utilizes low-intensity ultrasound (US) and sonosensitizers to generate cytotoxic ROS to induce tumor cell apoptosis [64,190,191,192]. The ultrasound can penetrate deeper into tissues compared to light, making it suitable for the treatment of deeper tumors. Ren et al. synthesized arginylglycylaspartic acid (RGD)-tagged carbon dots (RB-CDs@RGD) by synthesizing RB-CDs using glycine and rose bengal (RB) as precursors in a hydrothermal method and then conjugating RGD with RB-CDs via NHS/EDC coupling reaction [192]. The therapeutic efficacy of RB-CDs@RGD was assessed in C6 tumor-bearing nude mice. No significant weight changes between experimental and control groups were observed for 14-day treatments, indicating that RB-CDs@RGD and its combination with ultrasound did not induce toxicity. Notably, tumor growth was significantly inhibited for the RB-CDs@RGD+US group, whereas the control (i.e., saline) and RB-CDs@RGD groups exhibited significant tumor growth (Figure 15a,b), demonstrating the promising anti-tumor efficiency of the SDT using CDs.

#### 4.2.7. Gas Therapy

**Figure 16:**
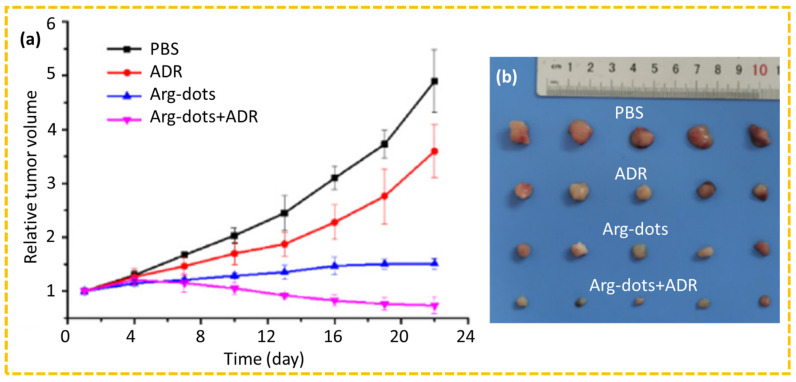
In vivo evaluation of tumor growth inhibition in MCF-7/ADR tumor-bearing mice following treatment with gas therapy: (**a**) the tumor volume growth curves up to 22 days and (**b**) photographs of excised tumors from each treatment group after 22 days [65].

The gas therapy employs endogenous gasotransmitters (i.e., NO, H_2_S, and CO) to modulate various biological functions within the tumor microenvironment [65,193,194,195]. To date, the L-arginine (Arg) CD study remains the primary example of CD-mediated gas therapy in animal models [65,195]. Liu et al. developed L-Arg-based CDs (Arg-dots) using L-Arg as the precursor in a microwave method [65]. The Arg-dots were shown to generate NO in H_2_O_2_-rich tumor environments. In vivo evaluations on human female breast cancer adriamycin (ADR) drug-resistant cell line (MCF-7/ADR) tumor-bearing mice revealed that the Arg-dots group exhibited a significant anti-tumor efficacy compared to the control (i.e., PBS) and ADR groups, with the Arg-dots+ADR group showing further suppressed tumor growth over 21 days (Figure 16a,b). This study demonstrates the efficacy of gas therapy on cancer and highlights the superior effectiveness of the combined treatment of Arg-dots and ADR relative to ADR and Arg-dots alone.

## 5. Conclusions and Future Perspectives

CDs have emerged as potential candidates for cancer theranosis due to their easy synthesis and surface modification, low toxicity, good water solubility, tunable fluorescence, good photostability, and various therapeutic properties such as PTT and PDT. Owing to the aforementioned outstanding properties, CDs have received significant attention as alternatives to dyes and QDs in the field of biomedical imaging. In this review, we explored the recent advancements of CDs, emphasizing their potential use in cancer theranosis. We discussed various surface modification methods of CDs to decorate their surface with various functional molecules for cancer theranosis, in vitro and in vivo toxicity to demonstrate their suitability for biomedical applications, and then, a key focus was placed on discussing their in vitro and in vivo applications to cancer theranosis, including drug delivery, PDT, PTT, PTT+PDT, and siRNA therapy.

As reviewed here, CDs have shown promising cancer theranostic properties in vivo and in vitro. However, several challenges must be addressed before the clinical application of CDs. Most current CDs exhibit absorption and emission in the visible region, which limits their use in deep tissue imaging and therapy. To overcome this limitation, there is an urgent need to develop CDs with strong absorption and emission in the NIR region. The NIR CDs can be used to improve tissue penetration and reduce background interference from autofluorescence. In addition, although CDs are known for their low toxicity, long-term in vivo toxicity studies (>1 year), including immunological assays, will be needed to confirm their safety for clinical translation. In addition, the scalable synthesis method of CDs with controlled particle size and a simple purification procedure is not currently available and thus needs to be developed to enhance commercial applications of CDs. In addition, CDs can be photodegradable, and thus, experimental conditions that provide no photodegradation under light irradiation need to be investigated for safe applications of CDs in PDT and PTT.

The previous studies support the idea that the future use of CDs in cancer theranosis is highly promising. With continued research and interdisciplinary collaboration between material scientists, chemists, biologists, and medical professionals, CDs may pave the way for clinical translation in cancer theranosis in the future.

## Figures and Tables

**Table 1:** Comparison of CDs with other nanocarriers.

Nanocarrier	Size (nm)	Advantage	Limitation	Ref.
CD	<10	- Easy, cheap, and green synthesis - Easy surface modification - Water soluble - Stable photoluminescence - Low toxicity	- Controlled synthesis of uniform CDs remains challenging - Long-term biosafety is not fully established	[30,125]
Micelle	5–100	- Enhances solubility of hydrophobic drugs - Shields degradation and reduce systemic toxicity of drugs - Biocompatible	- Poor water solubility of small-sized micelles - Poor physical stability in vivo	[126,127,128]
Liposome	50–150	- Carry both hydrophilic and hydrophobic drugs - Biocompatible - Protects drugs from degradation - Prolong circulation time to enhance passive tumor targeting	- Physical instability - Drug leakage before reaching the target - Limited drug loading capacity	[129,130]
Nanogel	<200	- High drug loading capacity - Biocompatible	- Polydispersity of polymers - Poor renal clearance	[131]
Polymeric nanoparticle	10–1000	- Controlled drug release - biocompatible - Possible surface functionalization	- Low scalability - Low reproducibility - Variability in drug release profiles	[132,133]

## Data Availability

No new data were created or analyzed in this study. Data sharing is not applicable to this article.
